# Nonribosomal Peptide Synthesis Definitely Working Out of the Rules

**DOI:** 10.3390/microorganisms10030577

**Published:** 2022-03-07

**Authors:** Matthieu Duban, Stéphane Cociancich, Valérie Leclère

**Affiliations:** 1 Université de Lille, Université de Liège, UMRT 1158 BioEcoAgro, Métabolites Secondaires d’origine Microbienne, Institut Charles Viollette, F-59000 Lille, France; matthieu.duban@univ-lille.fr; 2 CIRAD, UMR PHIM, F-34398 Montpellier, France; stephane.cociancich@cirad.fr; 3 PHIM, Université Montpellier, CIRAD, INRAE, Institut Agro, IRD, F-34398 Montpellier, France

**Keywords:** nonribosomal peptide synthetase, modular megaenzyme, secondary metabolites, natural products, assembly lines, NRPS

## Abstract

Nonribosomal peptides are microbial secondary metabolites exhibiting a tremendous structural diversity and a broad range of biological activities useful in the medical and agro-ecological fields. They are built up by huge multimodular enzymes called nonribosomal peptide synthetases. These synthetases are organized in modules constituted of adenylation, thiolation, and condensation core domains. As such, each module governs, according to the collinearity rule, the incorporation of a monomer within the growing peptide. The release of the peptide from the assembly chain is finally performed by a terminal core thioesterase domain. Secondary domains with modifying catalytic activities such as epimerization or methylation are sometimes included in the assembly lines as supplementary domains. This assembly line structure is analyzed by bioinformatics tools to predict the sequence and structure of the final peptides according to the sequence of the corresponding synthetases. However, a constantly expanding literature unravels new examples of nonribosomal synthetases exhibiting very rare domains and noncanonical organizations of domains and modules, leading to several amazing strategies developed by microorganisms to synthesize nonribosomal peptides. In this review, through several examples, we aim at highlighting these noncanonical pathways in order for the readers to perceive their complexity.

## 1. Introduction

Thousands of microbial secondary metabolites display large structural biodiversity and, consequently, a broad range of activities that can be exploited in different areas such as plant, animal, and human health. Since a few years, it has become relevant to identify new bioactive compounds for applications in a One Health context where ecologically and medicinally important nonribosomal peptides (NRPs) may play key roles. NRPs are built up by multifunctional mega-enzymatic complexes called nonribosomal peptide synthetases (NRPSs) that work in a thiotemplate-based sequential manner as assembly lines [1,2]. Due to their modular organization, the size of NRPSs is variable, but some of them can be exceptionally large (up to over a megadalton) and are therefore encoded by giant genes or groups of genes that, together with the cell surface protein-encoding genes, are considered as among the biggest in the microbial world and more generally in nature [3]. If NRPSs do catalyze the formation of peptidic (amide) bonds between amino acid monomers, their polyketide synthases (PKSs) counterparts form with a similar scheme of carbon-carbon linkages of aryl acid moieties, leading to the modular synthesis of polyketides (PKs) [4]. Interestingly, some genes do encode hybrid PKS-NRPSs resulting in the production of hybrid PK-NRPs.

The extreme structural diversity of the NRPs is mainly due to the ability of NRPSs, according to intrinsic characteristics, to incorporate nonproteinogenic amino acids in the final peptide, as well as to the action of tailoring enzymes. The latter is usually encoded within the NRPS biosynthetic gene clusters (BGCs) and perform structural modifications such as hydroxylations, glycosylations, or formylation, for instance, on the neosynthesized peptides. Since the gilded age of antibiotics in the 1940s, the discovery of new secondary metabolites occurred mostly through bioassay-based approaches. More recently, however, because of multiple rediscoveries and courtesy of the availability of exponentially increasing genomic data, the historical bioassay-based screening has slowly but surely been outcasted by the genome mining strategy. As a result, in silico analyses of thousands of available microbial genomes allow us to pinpoint NRPS BGCs, including some being cryptic or silent in laboratory conditions, therefore expanding our perception of the metabolic potential of microbes [5,6]. Concomitantly, bioinformatics tools have been developed, allowing researchers to shed light on new NRPs and to make predictions regarding their partial or complete structures [7,8,9]. Although biological assays will always be required to unravel the structure, function, and activity of newly discovered compounds, the genome mining strategy results in a considerable time saving compared to the bioassay approaches, especially avoiding the rediscovery of already known NRPs. Still, it needs continuous improvement of bioinformatics accuracy to predict the metabolic potential of the microorganisms. The discovery of new NRPs will therefore benefit from a better understanding of their biosynthetic pathways, especially since the biosynthetic processes involved in the nonribosomal peptide synthesis appear to be sometimes less canonical than initially thought. Hence, this review aims at providing examples of the microbial metabolic diversity found in nonribosomal peptide synthesis pathways with a focus on the “out of the rules” noncanonical NRPSs world. Nevertheless, this review is mostly limited to multimodular linear thiotemplate NRPSs, with a very few exceptions concerning some stand-alone module thiotemplate NRPSs [2,10].

## 2. Canonical Rules for Nonribosomal Synthesis

The NRPS assembly lines select and condensate step by step amino acids to build up peptides. The process strongly relies on the modular architecture of an NRPS, where each module stands as a structural block catalyzing the stepwise incorporation of one monomer (or building block) into the nascent peptide [11,12]. The modules represent repeating units of the enzymatic template for the production of overwhelming structural biodiversity of microbial secondary metabolites [1]. Remarkably, the NRPS proteins of a complete assembly line may be encoded by several genes, sometimes included in operons in bacterial genomes. In this case, the correct biological pairing between the products of the genes in the operon to form a single, complete and coherent assembly line is achieved and controlled by stretches of 20–30 amino acids forming communication (COM) domains [13,14,15,16].

### 2.1. Modular Assembly Lines including Core Domains

**Figure 1:**
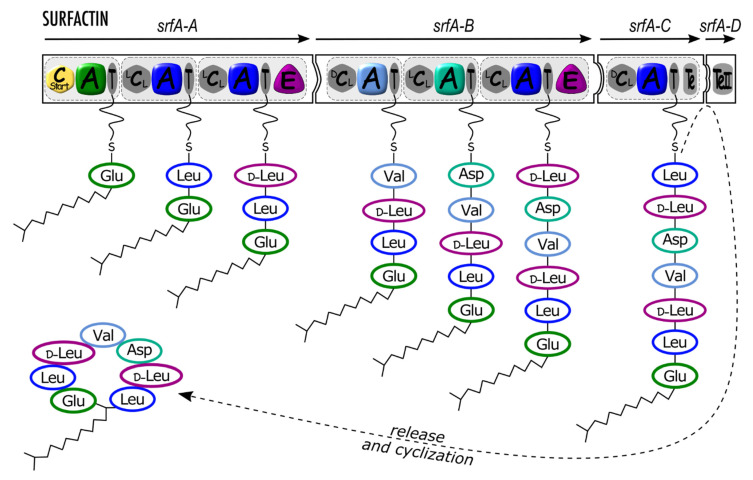
Surfactin synthetase architecture and schematic mode of synthesis. *srfA-A*, *srfA-B*, *srfA-C,* and *srfA-D* (arrows) are genes coding four synthetase proteins (black-framed puzzle pieces) organized in modules (light grey dotted rectangles with rounded corners) including domains as follows: Cstart (yellow hexagon): condensation starter domain; A (colored rounded square), adenylation domain; T (grey ellipse), thiolation domain (also called peptidyl carrier protein) carrying a phosphopantetheinyl (pPant) swinging arm; E (purple rounded triangle), epimerization domain; ^L^C_L,_ ^D^C_L_ (grey hexagon), condensation domain; Te, TeII (grey rounded rectangle), thioesterase domain. Incorporated amino acids (three-letter code) are circled with the respective A domain or E domain color.

To achieve nonribosomal peptide synthesis, the minimal enzymatic complexes contain four distinct domains called core or essential domains (Figure 1) [12]. The first one is the adenylation (A) domain. A domains are approximately 550 amino acids long and are members of the ANL (acyl-CoA synthetases, NRPS adenylation domains, and luciferase) superfamily. Their catalytic site includes a binding pocket able to receive the monomer that will be activated by an adenylation reaction with an ATP molecule. A striking feature of the nonribosomal synthesis is that the building blocks introduced into the peptides are not only proteinogenic amino acids because A domains can recruit nonproteinogenic amino acid monomers including, for instance, L-, D- and α-, β- or δ-amino acids as well as 2-aminoisobutyric acid (Aib, present in peptaibols), hydoxyphenylglycine (Hpg, present in glycopeptides antibiotics) or dihydroxybenzoate (Dhb, found in many siderophores) [17], among others. To date, more than 500 of such monomers have been identified (see the NORINE website for an updated list, http://norine.univ-lille.fr, accessed on 8 February 2022) [18]. An A domain is characterized by the presence of 10 conserved motifs referred to as a1 to a10 and distributed along the 550 amino acid-long sequence [19]. The substrate-binding pocket of A domains is formed by 10 amino acids scattered between motifs a3 and a6, which represent the substrate specificity-conferring sequence [20,21,22]. The relationship between the nature of the substrate specificity-conferring sequences and the selectivity of the monomer recruitment has led to the description of the Stachelhaus or NRPS code [23]. Many efforts have been produced to develop bioinformatics tools enabling the in silico prediction of the most probable recruited monomer by each A domain. As an example, the NRPSpredictor tool now included in antiSMASH (https://antismash.secondarymetabolites.org, accessed on 8 February 2022) [6] based on support vector machines [24,25] uses the physicochemical properties of the residues of the binding pocket that stand eight angströms around the substrate to predict the nature of the substrate incorporated from the sequence of the corresponding A domain. Nevertheless, unlike the ribosomal genetic code, the NRPS code shows some relaxed options and is not yet fully deciphered.

Some promiscuity of A domains, leading to the co-production of variants, has been observed and probably represents a means of environmental adaptation as it allows the synthesis of several analogs according to the substrate present at a given time. The amonabactins synthesized by the *amo* operon by *Aeromonas hydrophila* illustrate this versatility as the A domain of AmoG indifferently selects and activates two aromatic residues (Phe and Trp) in a ratio depending on the relative concentrations of both substrates in the medium [26]. The flexibility of A domains leading to the structural diversity of variants has also been described for the biosynthesis of cyclic lipopeptides (CLPs) by diverse *Bacillus* and *Pseudomonas* strains [27,28,29]. The promiscuous specificity of the A domains of modules 2, 4, and 7 of the surfactin synthetase is at the origin of the structural diversity of the lipopeptides belonging to the surfactin family, whereas the structure of the variants produced by several strains of the genus *Bacillus* influence their physicochemical properties and biological activities, and diversify their potential applications [29]. Another example lies within the maremycins biosynthetic pathway in *Streptomyces* sp., in which the A domain of MarQ adenylates with the same efficiency Cys and methyl-Cys, and to a lower extent Ser, allowing the production of major and minor compounds [30]. Detection of variants representing minor forms in mixtures will probably burst in the near future due to the availability of more sensitive methods and technologies for structural determination [31].

Once selected and adenylated, the activated monomer is then transferred and covalently tethered onto the approximately 80 amino acid-long thiolation (T) domain (Figure 1), also often referred to as PCP for peptidyl carrier protein. To be functional, T domains must carry a 4’-phosphopantetheine (Ppant) prosthetic swinging arm, which is beforhand brought by a stand-alone phosphopantetheinyl-transferase enzyme [32]. The flexibility of the arm ended by a thiol SH group is essential for the functioning of the NRPS since it shuttles the growing peptide along the assembly line [33].

The third core domain is the approximately 450 amino acid-long condensation (C) domain catalyzes the peptide (amide) bond formation between the monomers carried by its neighbors T domains. Different subtypes of C domains have been identified related to the nature of the condensated monomers [34]. Thus, ^L^C_L_ domains catalyze the formation of a peptide bond between two L-monomers while ^D^C_L_ domains condensate a D-monomer at the C-terminus of the growing peptide with an L-monomer loaded onto the following module (Figure 1). The classification of ^L^C_L_ and ^D^C_L_ make them predictable using bioinformatics tools such as NaPDoS (available at https://npdomainseeker.sdsc.edu, accessed on 8 February 2022) [35]. Noteworthy, although C domains do not possess substrate specificity pockets as in A domains, it has been shown that they participate to some extent in a kind of proofreading process that prevents them from performing the condensation reaction if, for instance, some required modifications of the T-bound aminoacyl thioester are missing, as is the case for glycopeptide antibiotics biosynthesis [36,37].

Finally, in a functional canonical assembly line, a terminal thioesterase (Te) domain catalyzes the release of the neoformed nonribosomal peptide from the enzyme complex. The peptide can be released by hydrolysis to produce linear compounds such as ACV (aminoadipic acid-cysteine-valine), the precursor of the famous antibiotic penicillin [38], or an intramolecular reaction can produce a cyclic peptide (Figure 1). Different cyclization strategies related to the three-dimensional architectures of Te domains are described [11]. Thus, a broad range of head-to-tail (e.g., tyrocidin) and sidechain-to-tail (e.g., bacitracin) macrocycles of various ring sizes can be produced. The latter structure is sometimes referred to as « partial cycle » in the Norine database [18].

### 2.2. Secondary Domains

The incorporated amino acids/monomers can be modified during the nonribosomal synthesis by enzymatic activities harbored by secondary domains (also referred to as auxiliary, accessory, and even ancillary, domains in the literature) [39,40,41,42,43] that take full part in the NRPS complexes. Secondary domains catalyzing modification reactions such as epimerization, methylation, or formylation have been described [1,44,45]. The monomer modifications, especially epimerization, methylation, and cyclization, are generally considered as structural features leading to increased resistance of these secondary metabolites preventing proteolytic degradation [44].

By far, the most frequently encountered secondary domain among NRPS is the epimerization (E) domain, which converts an L-form monomer into its D-form through isomerization of its α-carbon. Statistics based on Norine data indicate that nearly half of the referenced peptides contain at least one D-monomer [18]. When present in a given module, the E domain follows the T domain and catalyzes the epimerization of the monomer within the growing peptide tethered on it. Consequently, a C domain following an E domain belongs to the subtype ^D^C_L_ (Figure 1). A rare example where a D-monomer is directly selected by an A domain after epimerization by a *trans*-racemase is the cyclosporin A. The racemase encoded by *simB* is able to convert L-Ala into D-Ala, which is further adenylated by the first A domain of the NRPS encoded by *simA* [46].

**Figure 2:**
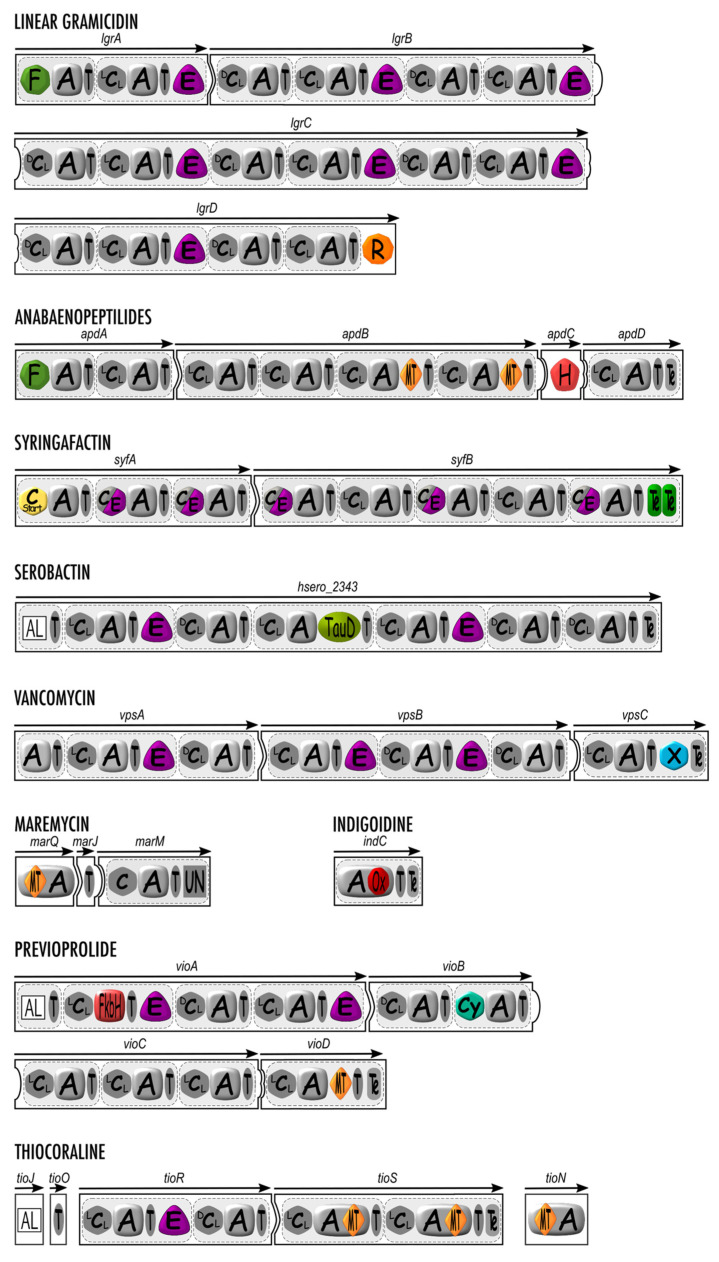
Secondary domains involved in nonribosomal peptide synthesis: structural organization of linear gramicidin, anabaenopeptilides, syringafactin, serobactin, vancomycin, maremycin, indigoidine, previoprolide, and thiocoraline synthetases containing secondary domains with monomer modification enzymatic activity. Canonical domains are depicted in grey, while secondary domains are highlighted with color. A, adenylation domain; C (^L^C_L_, ^D^C_L_, or C_start_), condensation domain; C/E, dual condensation and epimerization domain; T, thiolation domain; F, formylation domain; E, epimerization domain, R, reductase domain; MT, methyltransferase domain, H, halogenase domain; TauD, aspartate hydroxylation domain; X, X domain; Ox, oxidation domain; FkbH, FkbH domain; Cy, cyclization domain; UN, domain with unknown function; AL, Acyl-CoA ligase. The white squared domain is related to PKSs.

Another type of secondary domain is the methylation (MT) domain that catalyzes the transfer of a methyl group from S-adenosylmethionine (SAM) to the carbon or nitrogen atom of the peptide bond for methylation of the peptide backbone. N-MT and C-MT domains can be discriminated according to their sequence, allowing their identification by bioinformatics [47]. Generally, the MT domain is located upstream of the T domain on which the target residue is covalently linked. N-methylation occurs in bacterial NRPs such as anabaenopeptilides (Figure 2) and is a frequent feature in fungal NRPs such as aureobasidin or cyclosporin [48]. Notably, seven out of the 11 amino acids from the immunosuppressive agent cyclosporin are methylated. In this case, N-methylation plays a critical role in the biosynthesis mechanism and is a structural requirement for maintaining an active conformation of the peptide [49].

Formylation (F) domains are often listed among the NRPS secondary domains [1,44,45]. However, these domains are very scarcely observed in nature. To date, only three NRPs bearing an N-formylation due to a F domain (always found in the initiation module) have been described. F domains have been found in the initiation module of the anabaenopeptilides [50], the linear gramicidin [51], and the kolossin A [52] assembly lines (Figure 2). F domains in NRPSs display high similarity to the methionine-tRNA-formyltransferase allowing a cap protection of the bacterial ribosomal proteins. The N-formylation by F domains occurs once the amino acid is loaded onto the next T domain.

All these modifications can be catalyzed by secondary domains acting in *cis* during the assembling of the peptide but may also result from external tailoring enzymes acting in *trans* while intermediates are still covalently tethered onto the NRPS or post assembly after the release of the peptide. The tailoring enzymes are usually, but not exclusively, encoded by genes present in the BGCs. More specifically, tailoring enzymes with methylation or formylation activities are frequent in nonribosomal secondary metabolite biosynthetic pathways. For instance, BGCs for both the *Burkholderia* siderophores malleobactin and ornibactin and some *Pseudomonas* pyoverdines contain genes encoding enzymes with a formyltransferase activity that are located downstream the NRPS genes [53,54]. While F domains acting in *cis* only formylate the N-terminal amino acid, the formyltransferases catalyze the modification of a monomer located inside the peptide moiety and therefore account for the higher prevalence of formylated secondary metabolites described in the Norine database compared to what is expected with the action of F domains.

### 2.3. Modes of Biosynthesis

Modes of nonribosomal peptide biosynthesis have been classified into three types: linear (type A), iterative (type B), and nonlinear (type C) [1]. The latter is like a tote bag as it includes non-strictly linear and non-strictly iterative modes, whatever the order in the use of the modules and domains [55].

The linear mode of biosynthesis refers to the collinearity between the order of the modules in the nonribosomal assembly line and the sequence of the incorporated monomers in the final peptide [1,44,55]. That means that the number and order of modules in the NRPS coincide with the number and order of monomers in the peptide. It is important to distinguish between the linear mode of biosynthesis and the final structure of the peptide that can be either linear as the penicillin precursor ACV [38] and linear gramicidin [51] or head-to-tail macrocyclized as surfactin (Figure 1) or also displaying sidechain-to-tail cycles as fengycin [56] and most of the *Pseudomonas* lipopeptides [57].

The most famous iterative mode of biosynthesis described is the one driving the synthesis of gramicidin S. This antibiotic produced by *Brevibacillus brevis* is a cyclic decapeptide while its biosynthetic pathway, consisting of two proteins encoded by an operon, only contains a total of five modules. Here, the Te domain has a key role in allowing the enzyme to repeat twice the process of synthesis hitherto called the iterative mode of biosynthesis [11,58]. An iterative mechanism is usually found in the synthesis of nonribosomal siderophores such as enterobactin or bacillibactin [59]. Indeed, it allows the release of trimers, a structure necessary for efficient iron chelation.

The type C mode of biosynthesis, nonlinear, gathers all the modes that do not fit within types A and B. In this class, a broad range of NRPSs is found irrelevant to their module and domain organization. Besides the promiscuity of A domains and the cyclization due to Te domains, the nonlinear mode of biosynthesis is probably responsible for a great part of the structural diversity of the NRPs as it mainly leads to branched structures. In some type C NRPSs, not only modules are used iteratively, but sometimes domains only are iteratively operated. As an example, in the mannopeptimycin NRPS, which exhibits a [C-A-T-E-C-A-T-C-T-E] domain organization, the second A domain proposes the same monomer to both subsequent T domains [60]. The type C NRPSs are characterized by an unusual arrangement of the core domains leading to unusual internal cyclizations or branch-point syntheses [55]. From these assembly lines, it is more difficult to predict the products from the NRPS architecture as it relies on an unexpected logic. While the first characterized NRPS assembly lines were of linear type, NRPS of type C was regarded as an exception. Nowadays, considering the evolution of the tools allowing genome mining and the prediction of the structure of the peptides from the sequence of NRPSs, it seems that the type C biosynthesis becomes major and leads to the discovery of more and more amazing pathways as described below.

## 3. Domains Working Out of the Canonical Rules

During the last decades, the discovery of an exponentially increasing number of nonribosomal peptides together with the characterization of the architecture of the corresponding NRPS biosynthetic lines highlighted that numerous NRPSs work out of these basic canonical rules, leading to yet-superior structural biodiversity of the secondary metabolites produced.

### 3.1. C Domains Working Differently

Besides their main activity, some C domains have been characterized as bifunctional. The first example is the characterization of dual condensation/epimerization (C/E) domains (Figure 2) initially found in the *Pseudomonas* arthrofactin synthetase [61]. Indeed, despite the presence of six amino acids in D-configuration over the 11 residues constituting the arthrofactin lipopeptide, no E domain has been identified within the NRPS. Later, it was demonstrated that C domains are replaced by C/E domains that have a dual catalytic activity with timing where epimerization precedes the condensation to the next amino acid [61]. The presence of C/E domains was further generalized in all NRPSs assembling lipopeptides for bacteria belonging to genera *Pseudomonas* [28] (Figure 2), *Burkholderia* [53,62], and *Xanthomonas* [63]. It is interesting to underline that when a single strain is able to produce several nonribosomal peptides, only lipopeptide NRPSs harbor C/E domains while the other NRPSs may have E domains followed by ^D^C_L_ domains [53].

C domains may be replaced by heterocyclization (Cy) domains, which catalyze both the peptide bond formation and subsequent cyclization of Cys, Ser, and Thr to form a thiazoline, oxazoline, or methyloxazoline ring, respectively [64]. Cy domains are evolutionary related to C domains [34] (Figure 2). They are responsible for the two-step condensation/cyclodehydratation on the growing peptide tethered onto a T domain, as it has well been deciphered for the bacillomycin synthetase [64,65].

More recently, a Te domain was identified to harbor two catalytic activities. For the first time, a Te domain was shown to catalyze epimerization of the final monomer introduced into the nocardicin peptide, a β-lactam antibiotic produced by *Nocardia* [1]. In vitro reconstitution of the excised Te domain together with detailed biochemical characterization of a series of potential peptide substrates showed that the Te domain ending the nocardicin NRPS is responsible for catalyzing epimerization of the C-terminal pHPG residue to D-configuration before releasing nocardicin G as a final peptide [66]. To our knowledge, no Te/E has been described to date for any other assembly line.

**Figure 3:**
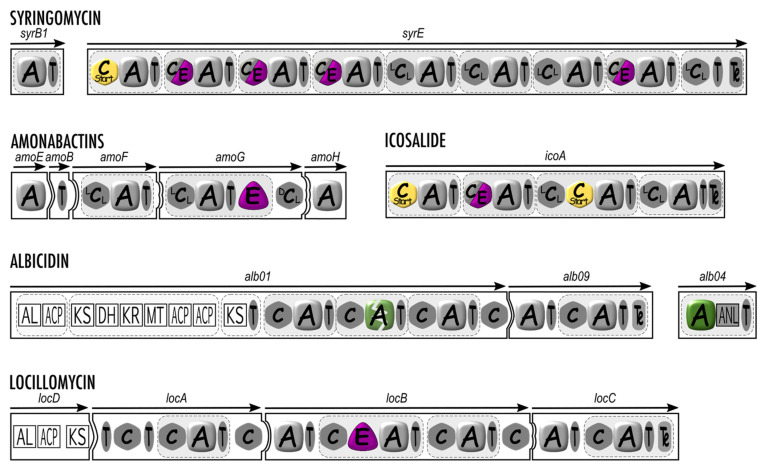
Structural organization of syringomycin, amonabactins, icosalide, albicidin, and locillomycin synthetases with an “out of the rules” mode of biosynthesis. A, adenylation domain; C (^L^C_L_, ^D^C_L,_ or C_start_), condensation domain; C/E, dual condensation and epimerization domain; T, thiolation domain; E, epimerization domain; MT, methyl transferase domain; AL, acyl-CoA ligase domain; ACP, acyl carrier protein; KS, ketosynthase domain; DH, dehydrogenase domain; KR, ketoreductase domain; ANL, α-ANH-like domain. The nonfunctionnal second A domain of albicidin synthetase is depicted with a green broken rounded square. The white squared domains are related to PKSs.

Cyclic lipopeptides (CLPs) are major biosurfactants produced by bacteria, displaying activities with very interesting applications [5,57,67]. The N-terminal acylation of CLPs is usually achieved by coupling a fatty acid onto the first amino acid of the peptide moiety assembled by the NRPS. The reaction of lipoinitiation is catalyzed by a C domain subtype starting the assembly line and C starter (Cstart) domain (Figure 1, Figure 2 and Figure 3) [34], whereas the initiation module of the other biosynthetic assembly lines usually starts with an A domain. A Cstart domain is present as the first domain of the initiation module of CLPs produced by *Bacillus* [56], *Pseudomonas* [28], *Burkholderia* [53], and *Xanthomonas* [63]. Unlike other CLPs, locillomycin and the different iturins are synthetized by a PKS-NRPS hybrid complex in which no Cstart domain is found because the fatty acid is introduced by the acyl ligase (AL) domain of the PKS module [56,68,69,70]. Moreover, Cstart domains are also found starting the biosynthetic assembly lines of compounds with lipopeptidic nature but not belonging to the CLP super family because of their low surfactant properties. Thus, antibiotics of clinical importance related to the daptomycin family, such as daptomycin, CDA, friulimycin, or A54145, are synthesized by NRPSs with a Cstart domain as the first domain [71].

More recently, X domains have been described, found exclusively in the termination module of NRPS assembly lines for the aglycone precursors of glycopeptide antibiotics (GPAs), more precisely between the last T and the Te domains [72] (Figure 2). GPAs are heavily modified heptapeptides displaying several intramolecular cyclizations as well as glycosylation, acylation, sulfation, and halogenation. Including teicoplanin, balhimycin, and vancomycin as representative members, they stand as the last clinical antibiotics efficient against the methicillin-resistant *Staphylococcus aureus* [73]. X domains are evolutionary related to C domains and most closely to ^L^C_L_-type C domains, but they are inactive as condensation domains due to the mutation of amino acids in their highly conserved catalytical site HHxxxDG motif, which has been shown to be essential for the formation of the peptidic bond and epimerization [34,45]. X domains have rather been shown to act as platforms for the recruitment of monooxygenases belonging to the cytochrome p450 superfamily, the latter being responsible for the cyclization of the T-bound nascent peptide via cross-linking of amino acid aromatic sidechains. As such, X domains are responsible for the *trans*-activation of tailoring enzymes [72]. Three cytochrome p450 enzymes for vancomycin, and four for teicoplanin, are sequentially recruited by the dedicated X domain in their respective biosynthetic lines to catalyze amino acid sidechains cross-linkings resulting in the formation of three or four, respectively, macrocycles and therefore providing aglycone molecules, which already possess the rigid three-dimensional structure that is responsible for the antibiotic activity of the GPAs [72,74,75]. A cristallography study showed that during the biosynthesis of teicoplanin, the multiple cytochrome p450 enzymes required for the macrocyclizations all interact in a sequential manner according to the cyclization status of the nascent peptide, with the same site on the X domain surface for which they compete. The X domain-cytochrome p450 enzymes interactions take place at a micromolar range and occur mainly through hydrogen bonds and salt bridges [72]. Notably, unlike other cytochrome p450 enzymes, the cytochrome p450 enzymes recruited by a GPA X domain bear a conserved Pro-Arg-Asp-Asp motif in the F-helix [75]. Finally, it is worthy to note that the X domain, together with a 120 amino acid extension close to the Te domain, is thought to originally represent an additional module in an ancestor GPA-producing NRPS [34]. While the remnant C domain of this module has been modified through evolution toward a cytochrome p450 recruitment platform (i.e., the X domain), the Te domain shares some sequence homology with an A domain [76].

### 3.2. Discovery of New Rare Secondary Domains

TauD domains are rare domains so far only found in NRPS assembly lines of some siderophores such as serobactins [77], cupriachelins, or taiwachelins [78] (Figure 2). Unlike siderophores relying on catechol or hydroxamic groups to chelate iron, they bear an unusual β-hydroxy-aspartic acid residue as they require a hydroxy-carboxylic functional group for Fe(III) coordination. During the biosynthesis of these siderophores, hydroxylation of aspartate is performed either by a discrete aspartyl-β-hydroxylating tailoring enzyme or/and through a TauD hydroxylating domain located within the NRPS, both displaying sequence homology with non-heme Fe(II) α-ketoglutarate-dependent dioxygenases such as the SyrP enzyme involved in syringomycin E biosynthesis [79,80]. TauD domains are always adjacent to L-Asp-selective A domains. The hydroxylation of aspartate takes place following the adenylation step as the Asp residue is tethered to the T domain, as shown by an analysis of the substrate specificity of the A domains for cupriachelin [78].

FkbH domains are rare domains found rather in PKS assembly lines. However, a few NRPS (or PKS/NRPS hybrids) assembly lines do contain such domains with significant sequence identity to glyceryl-transferases/phosphatases from the haloacid dehalogenase superfamily [81]. In NRPS modules, FkbH domains stand in place of A domains and perform the loading and dephosphorylation of D-1,3 biphosphoglycerate to afford a glyceryl moiety on a T domain, as is the case in the biosynthesis of the glycosylated lipopeptides cystomanamides [82] and the anticancer lead compound vioprolide [83] (Figure 2) both isolated from the soil myxobacteria *Cystobacter* sp. As a matter of fact, the vioprolide biosynthetic line does contain two unusual domains, i.e., a fatty acyl AMP ligase (FAAL) domain in module 1 (PKS) which recruits a fatty acyl, and the FkbH domain in module 2 (NRPS) (Figure 2). The latter allows the incorporation of a glycerate subsequently linked to the fatty acid via an unusual ester bond catalyzed by an ester bond-forming C (EBFC) domain [64].

Other domains displaying unusual or unknown functions are found scarcely throughout the literature. For example, an AKN domain, with sequence homology to adenylsulfate transferase, an enzyme responsible for the biosynthesis of PAPS (precursor for the sulfamate group of monobactams), has been identified in the initiation module of the sulfacezin assembly line [84]. In addition, the chondramides biosynthetic assembly line exhibits a phosphoenolpyruvate (PEP) domain at its very end (right after the Te domain), but the authors did not elaborate on a possible role for this domain [85]. Finally, a domain of unknown function has been identified at the N-terminus of the biosynthetic assembly line of the substituted vinylglycine AMB (L-2-amino-4-methoxy-trans-3-butenoic acid). Depending on the publications, this domain has been named either “?” domain [86], UN domain [30] (Figure 2), U domain [87], or Q domain [88].

### 3.3. Secondary Domains Nested within A Domains

Secondary domains are generally present as separate domains included within assembly lines. However, A domains, including an auxiliary domain (or a part of an auxiliary domain), have been identified with methyltransferase, monooxygenase, oxidase, or reductase activities and are sometimes described as bifunctional [42,89]. In this case, the secondary domain is generally nested between conserved sequences a8 and a9, as a loop region serves as an evolutionary insertion point of domains catalyzing amino acid modifications [1].

The most commonly interrupted A domains identified are those involved in N-methylation of peptide backbones. Thus, two A domains of TioS, an NRPS involved in thiocoralin synthesis, are interrupted by an MT domain (Figure 2) that methylates backbone amine nitrogens, with methylation probably occurring onto the T-attached amino acid rather than on the T-attached nascent peptide [89]. The same assembly line for thiocoraline biosynthesis also includes TioN with an A domain disrupted by an MT domain, capable of adenylating L-Cys before its S-methylation. The location of this interruption stands exceptionally between sequences a2 and a3 [42]. A similar insertion of an MT domain between motifs a2 and a3 is also found in the A domain of MarQ involved in maremycin synthesis [30] (Figure 2).

A flavin-dependent oxidation (Ox) auxiliary domain can be nested between sequences a8 and a9 of an A domain as in the monomodular NRPS IndC responsible for indigoidine synthesis. Indigoidine is a dimeric purple/blue pigment nonribosomally synthesized from L-Gln recruited by the A domain. Then, the Ox domain installs a carbon-carbon double bond after tethering onto the T domain or after macrocyclization and release [90].

### 3.4. Domains Ending an Assembly Line

Besides the canonical Te domains ending lines, some NRPSs include a supplementary discrete Te domain called type II Te (TEII) domain [58] as in the surfactin assembly line (Figure 1). TeII domains are referred to as having a proofreading role as they are able to regenerate a functional 4’phosphopantetheinyl arm of a misprimed T domain [91]. Their role is to avoid the release of misassembled peptides and improve the efficacy of the biosynthesis because they can hydrolyze off incorrect cofactor or peptidyl groups tethered on T domains [44]. For some NRPSs, a tandem of Te domains is directly included within the protein ending the assembly line. This unusual tandem architecture was identified in the termination module of LybB, one of both NRPSs involved in the biosynthesis of the depsipeptide lysobactin [92]. In this tandem, the first Te domain was shown to be exclusively responsible for the regio- and stereoselective macrocyclization of the undecapeptide, whereas the second Te domain solely catalyzes the release of the lysobactin from the synthetase [92]. The teixobactin NRPS also ends with tandem Te domains in which both Te domains were shown to be exchangeable and likely acting synergistically [93].

*Pseudomonas* strains produce a large variety of CLPs, currently classified in at least 14 groups [57]. Most of them are synthesized by assembly line carrying a tandem of Te domains in the termination module [94]. This includes orfamides and sessilin [95], massetolide, arthrofactin, viscosin, putisolvin, and tolaasin [96]. This peculiar feature has been used to identify putative CLP gene clusters by in silico analysis [97]. Interestingly, whereas peptin, factin, and mycin families may be co-produced by *P. syringae* strains, only the peptin and factin NRPSs display a tandem of Te, whereas mycin synthetases possess a single Te domain [28] (Figure 2 and Figure 3). The role of each of Te domain in a tandem architecture is not clearly established. Indeed, whereas in some cases, one Te catalyzes the release from the NRPS while the other one is involved in the cyclization of the lipopeptide, it is not possible to generalize the rule as there is no direct relationship between the presence/absence of a Te tandem and the production of a cyclic/linear lipopeptide [94].

Cyclization of nonribosomal peptides is a key step in their biosynthesis. In bacterial NRPSs, Te domains catalyze the peptide cyclization, whereas, in fungal cyclic peptide NRPSs, no Te domain is present. Indeed, in fungal NRPSs that produce cyclic peptides, each synthetase generally constituted of a single protein is ended by a C domain called the C-terminal (C_T_) domain that is responsible for the release and macrocyclization of the peptide. First deciphered for cyclosporin A, aureobasidin A, apicidin, ferrichrome, destruxins, and tryptoquialanin, this strategy seems to be universally employed by fungal NRPSs [48]. This feature was used to identify a BGC involved in the cyclochlorotin production by the *Talaromyces islandicus* fungus using specific signatures to mine genome sequences in silico [98,99].

Most NRPS assembly lines possess a canonical Te chain-terminating domain, which, as described above, allows detachment (and eventually cyclization) of the nascent peptide. However, some NRPS and PKS/NRPS assembly lines do rely on a terminal reductase (R) domain (Figure 2) to perform the release of the tethered peptidyl thioester. R domains show significative sequence homology to members of the short-chain dehydrogenase/reductase (SDR) superfamily, which are tyrosine-dependent oxidoreductases. As such, R domains possess a conserved Ser/Thr-Tyr-Lys catalytic triad. They also exhibit a conserved Rossmann-type NADPH-binding site since they use NAD(P)H as a cofactor. R domains perform 2e^−^ or 4e^−^ reductions to release the final product from the assembly line [100,101]. The first 2e^-^ reduction is the real release step and generates a free aldehyde intermediate, which generally interacts again with the R domain and undergoes a second reductive (4e^−^) reaction, which creates the final product bearing a primary alcohol group. The second reductive step mainly allows to prevent the accumulation in the cell of toxic aldehyde compounds, but it sometimes occurs that the final product is aldehydic, as is the case for the saframycin A precursor [102] or for the flavopeptins [103]. R domains are found in the myxochelins [104], myxalamids [105], and linear gramicidin [106] synthetases, for instance (Figure 2). Some R domains also perform head-to-tail imine macrocyclizations of the free aldehydes, such as during the biosynthesis of aureusimine [107] and nostocyclopeptides [108]. Notably, in some NRPS systems, the second reductive step is performed by a tailoring aldo/keto reductase enzyme, as illustrated by the examples of linear gramicidin [106] and bogorol [109].

R* domains are R-like domains lacking the critical Tyr residue within their catalytic site, which prevents them from performing 2e^−^ or 4e^−^ reductive operations. Instead, R* domains catalyze non-reductively intramolecular Dieckmann cyclizations, which result in the formation of tetramic acid scaffolds. Many examples are found in the literature, as, for instance, the fungal NRPs equisetin [110] and (pre)-tenellin [111].

## 4. Amazing Modes of Biosynthesis

### 4.1. How to Overcome the Lack of Functional A or C Domains

When deciphering NRPS organization into domains, some assembly lines seem to lack essential A domains, and one might then wonder whether they are fully functional. Characterization of the assembly line of syringomycin provided the first insight into the synthesis of *Pseudomonas* CLPs. The corresponding BGC spans over 37 kb in size and shows an unusual architecture. The NRPS consists of a major protein, SyrE containing eight complete modules and a ninth module lacking an A domain. Here, the ninth amino acid is provided by the separate NRPS stand-alone module encoded by *syrB1* located upstream of *syrE*. In fact, the last module is split on SyrB1 [A-T] and on the end of SyrE [C-T-Te] (Figure 3). During the synthesis, the eight first amino acids are condensated according to the SyrE template in a collinear mode. SyrB1 independently activates and loads Thr. Once tethered on the SyrB1 T domain, the Thr residue is chlorinated by the halogenase SyrB2 and transported by the shuttle protein SyrC to the last T domain of SyrE for condensation to the nascent octapeptide prior to cyclization [28,94,96]. The special feature of this nonlinear assembly line is that it contains two split modules for the incorporation of the last amino acid. Additionally, this process allows the chlorination of the Thr before its incorporation into the final peptide.

A slightly different situation concerns the biosynthetic assembly line of the hybrid PK/NRP potent DNA gyrase inhibitor and phytotoxin albicidin, for which the A domain is present but not functional. The biosynthetic machinery of albicidin involves noncanonical complementation in *trans* of an inactive A domain within the main assembly line by a stand-alone unusual [A-T] di-domain module (Figure 3). Indeed, the A domain expected by the collinearity rule to incorporate a cyano-L-Ala in the final peptide is not functional due to low sequence conservation in the core motifs required for the correct activation and adenylation of a monomer. Since the incorporation in albicidin of this rather unusual cyano-L-Ala cannot be mediated by the defective A domain, it has been proposed that this reaction is *trans*-complemented by the unusual [A-T] stand-alone module encoded by the separate gene *alb04* in the albicidin BGC [112]. Interestingly, ATP-PPi exchange experiments showed that the preferred substrate activated by the A domain of *alb04* is Asn. However, in *alb04*, the presence between the A and T domains of a 342 amino acid-long extension exhibiting sequence homology, including the SGGKD ATP-binding motif, to members of the adenosine nucleotide α-hydrolase (α-ANH-like) superfamily prompted the authors to propose the following scenario for the processing of L-Asn to cyano-L-Ala and its subsequent incorporation into the skeleton of albicidin: the α-carboxy acid moiety of Asn is adenylated and stored as a thioester, followed by phosphorylation of the sidechain amide oxygen and subsequent dephosphorylation leading to the formal elimination of a molecule of water [112]. A similar biosynthetic scheme is also observed in the albicidin-gemini molecule cystobactamid [113], for which it has recently been shown that the α-ANH-like domain (rebaptized as AMDH) sequence in the stand-alone [A-T] module performs both dehydration and aminomutation of L-Asn to form the L-iso-Asn monomer incorporated in the cystobactamid molecule similar to the cyano-L-Ala in albicidin [114].

Such *trans*-complementations of an inactive A domain within a multimodular assembly line by a stand-alone [A-T] module encoded by a separate gene is also found in the biosynthesis process of the antitumor agents ramoplanins [115], enduracidins [116], and naphthyridinomycins, although for the latter it is unclear whether the complementation occurs in *trans* or in *cis* [117]. Notably, unlike the albicidin/cystobactamid stand-alone module bearing the α-ANH-like extension at the C-terminus of the A domain, the stand-alone modules of enduracidins and ramoplanins do not exhibit this supplementary sequence but rather an N-terminal 300 amino acid-long extension with no substantial homology to any other known sequence. However, it has been shown that this N-terminal sequence is required for the *trans*-complementation to take place during the biosynthesis of ramoplanins [118].

### 4.2. Complex Nonlinear Modes of Biosynthesis

To date, the more complex mechanism described is probably the one leading to the co-production of amonabactins by *Aeromonas* species using an NRPS encoded by the amonabactin *amo*CEBFAGH operon. Amonabactins constitute a family of four variants of catechol peptidic siderophores thanks to a unique mode of biosynthesis with alternative, iterative and optional use of domains [26]. The relationship between the domain organization of the NRPS (Figure 3) and the structures of amonabactins was demonstrated by the construction of mutants [26]. The mode of biosynthesis was qualified as an alternative because of the flexibility of the A domain of AmoG able to recruit indifferently Phe or Trp residues. The iterative mode refers to the AmoE-AmoF part reacting twice to link the fragment [Dhb-Lys] onto Phe/Trp. Finally, the optional mode was proposed because the Gly residue is introduced in the Lys sidechain in only two over the four related amonabactins.

Icosalide is an unusual asymmetric two-tailed lipopeptide produced by different *Burkholderia gladioli* strains isolated from a range of sources, including lung infection, mushroom rot, and insect [119]. In silico analysis of the isocalide biosynthetic pathway revealed an unprecedented NRPS that incorporates two β-hydroxyacids onto the same peptide chain using two Cstart domains embedded into modules 1 and 3 [120]. The NRPS, organized into four modules, contains four A domains, two of which are predicted to be specific for Leu and two for Ser. It also contains five condensation domains, including two Cstart, one C/E, and two ^L^C_L_. In vitro experiments based on heterologous production of the [Cstart-A-T] module 3 in *E. coli*, converted into holoform by a 4’phosphopantetheinyl transferase, demonstrated that the Cstart domain embedded in module 3 was able to acylate the following Ser residue. This experimental result fully supports the model of a unique assembly mechanism involving two distinct chain initiation events on a single NRPS subunit [119].

Surfactins, fengycins, and iturins are well-known families of CLPs produced by *Bacillus subtilis* strains [56]. Locillomycins are members of a novel family of cyclic lipopeptides containing nine amino acids forming the peptide moiety [70]. They are synthesized by a hybrid PKS-NRPS constituted of one PKS module and six NRPS modules starting with the following organization: [ACS-T-KAS][T-C-T-C-A-T-C]. This is a unique assembly line with NRPS modules 2, 3, and 4 used twice, whereas the first module and the two last modules are used only once. Moreover, several domains are skipped or optionally selected. First, the activated fatty acid loaded onto the T domain of the PKS module is condensed on the Thr activated by the A and T domains of the NRPS module 1, skipping the KAS domain and both subsequent T domains, with the optional use of one out of both C domains upstream of the first A domain. Then, the process continues in a canonical manner until module 4, and iterative use of modules 2, 3, and 4 leads to the lipopeptide containing seven residues. Finally, this hepta-lipopeptide is elongated to the final nona-lipopeptide by the two last modules before being released and macroclyzed by the Te domain [70].

The ε-poly-L-Lys synthetase is a membrane-bound unusual NRPS exhibiting a single noncanonical module containing an A and a T domain followed by three C-like domains referred to as C1, C2, and C3 [121,122]. This enzyme, initially discovered in *Streptomyces* strains, is able to produce the small cationic isopeptide ε-poly-L-Lys, one of the two only amino acid homopolymers known in nature. ε-poly-L-Lys is a polymer of 25–35 Lys residues bound together between their α-carboxylic and ε-amino groups, which encompass antimicrobial activity and, due to high stability and low allergenic properties, is widely used as a food preservative [121]. The biosynthetic process yielding ε-poly-L-Lys is as follows: L-Lys is specifically adenylated by the A domain and subsequently transferred to the T domain. The C1, C2, and C3 domains then catalyze bond formation between the covalently linked L-Lys extending unit on the T domain and a freely available L-Lys residue. This step is iteratively repeated with the free neosynthesized Lys dimer (or subsequently trimer, tetramer, etc.) used as an acceptor for a new L-Lys residue bound on the T domain [121,122]. None of the three C domains of the ε-poly-L-Lys synthetase do possess the usual catalytic site required for a condensation reaction but rather rely on an acyl ligase activity to perform the bond between Lys residues [122,123].

## 5. Conclusions/Outcomes

Since the discovery of nonribosomal peptides and the characterization of their synthetases and how the latter basically work, numerous examples have been identified and characterized, as shown by the regular and exponential increase in related papers published in the literature. Indeed, the number of papers about enzymatic or nonribosomal biosynthesis of peptides has grown from a very few dozen covering the 1960s and 70s to an average of a couple of monthly papers in the 90s and to almost a daily paper in 2021. This can be explained by the attractive applications of most of the NRPs in environment health (i.e., biosurfactants used in bioremediation), in plant health (i.e., lipopeptides with antifungal activities), as well as in veterinary and human health (i.e., antitumor and numerous antibiotics). Moreover, because of the emergency in identifying new solutions to overcome multidrug resistance, all tools and papers leading to spreading knowledge on the biosynthetic pathways of such compounds will be helpful.

As described in this review through relevant examples, a continuously increasing number of NRPSs are shown to not follow the canonical rules initially described. Beyond the noncanonical pathways described in this review, an additional level of complexity can be reached with compounds synthesized by stand-alone modules or synthesized through *trans*-esterification of two NRPs synthesized by two different NRPSs [124], for instance. The multiplication of these noncanonical examples should alert when genome mining is performed, especially when the expected/predicted product by the currently available bioinformatics tools, all based on the canonical rules, is not found in the growth supernatant. Therefore, the scientific community should be aware that automatic predictions performed by bioinformatics tools are subject to caution and that manual corrections based on the examples provided in this review might be mandatory.

## Data Availability

Not applicable.
